# Supplementary material for: Impact of layered behavioral, socio-economic and school-based interventions on selected behavioral and biomarker indicators among adolescent girls and young women in Uganda
Source: PLOS Glob Public Health. 2025 Jun 24;5(6):e0004819. doi: 10.1371/journal.pgph.0004819 (PMC12186927; doi:10.1371/journal.pgph.0004819)
Supplement: S1 Text — (DOCX) [file pgph.0004819.s001.docx]

**Detailed description of the Global Fund intervention package for AGYW**

1. **Interventions for out-of-school AGYW**. These interventions were implemented by TASO, with technical support from the Ministry of Health and the Ministry of Gender, Labor and Social Development, working closely with sub-recipient implementing organizations (sub-recipients). Out-of-school AGYW who received SBCC along with the other interventions were considered to have received a comprehensive package of interventions.
2. ***Social and behavior change communication*** (SBCC): The focus of SBCC is to equip girls with knowledge and skills intended to reduce the risk of new HIV infections, early marriages, teenage pregnancy, preventing STIs, and early sex among AGYW. SBCC activities target all AGYW aged 10-24 years living in the intervention communities. Girls are exposed to SBCC activities either as part of other interventions (for instance, girls enrolled in vocational skilling interventions have to attend weekly SBCC sessions for three months (before, during and after the training), extending for up to one year) or as stand-alone activities. Most of the girls are reached through stand-alone activities.

Small SBCC sessions (with up to 20 girls) are conducted by mentor mothers, members of village health teams (VHTs), and members of the district health team (DHT) while large meetings are conducted by project facilitators. VHTs talk to the girls during the market days with loudspeakers while DHT members offer comprehensive HIV packages before the tournaments. SBCC activities are conducted using a variety of approaches including community outreaches, community dialogue meetings, community-based radio talk panel discussions, out-of-school sports tournaments with link to sexual and reproductive health/HIV services, drama competitions for out-of-school AGYW, and information, education and communication (IEC) materials distribution for both in-school and out-of-school AGYW. SBCC messages included HIV and other STI prevention messages, gender-based violence prevention, and sexual and reproductive health issues (teenage pregnancy, menstruation hygiene management), among other areas. While all sub-recipients indicated that the messages are age-stratified, we did not see any evidence of a standard/comprehensive SBCC communication brief with key messages and their support points for use during SBCC sessions. Thus, it seems that a generalist mass-media approach (intended to reach ‘masses’) was used to reach AGYW with messages, usually in large groups, e.g., at sporting events.

1. ***Vocational skills training****:* Vocational skills training is aimed at training the AGYW in some form of skill that they can use to earn money so as not to engage in HIV risk behavior. The tuition fees are fully paid for by the sub-recipients but the girls are required to transport themselves to the training venue and provide their own personal requirements. Vocational skills training is offered to out-of-school vulnerable AGYW 15-24 years (with at least primary seven level of education) who are supported with tuition fees to join a vocational institution to acquire practical skills as a way building capacity for self-employment and engaging in gainful employment. Prior to enrolment, girls are profiled by the village leadership, working closely with members of the responsible sub-recipient organization. Profiled girls are screened at sub-county and district levels to confirm their vulnerability, with a final list of beneficiaries approved by TASO. The district is involved in identifying and enrolling the girls into district based vocational institutions which must be registered at the district and accredited to offer the necessary courses chosen by the girls.

Vocational skills training lasts for three months (with the exception of TASO-Bukwo where the training lasts six months), led by pre-qualified local artisans approved by TASO. Upon completion, all trainees have to undergo a mandatory internship (industrial training) of one-and-a-half months, and then sit for the Directorate of Industrial Training (DIT) exams. Girls are guided to choose a marketable course, based on circumstances obtaining in their respective districts, and can choose from a variety of courses including tailoring, hair dressing, motor vehicle mechanics, carpentry, or catering. It is important to note that, in a few instances, the course that the girls want may not be offered at the vocational training institute where they have been enrolled; in that case, they would have to choose from the available courses. Successful trainees, who have passed the DIT exams, receive completion certificates and are provided with start-up kits, including sewing machines, basic wood-working machines, and mechanical tool boxes, among others, depending on the course that girls participated in. As per protocol, all AGYW enrolled for vocational skills training receive SBCC messages and access to a comprehensive package of services including screening for GBV, and access to HIV testing, sexual and reproductive health, and family planning services, among others, by health workers and counselors from health facilities within the community.

1. ***Enterprise development assistance (EDA)***: EDA is intended to support girls with business skills and monetary support so as to make them economically empowered and hence reduce their involvement in risky behavior because of money. The intervention targets AGYW aged 15-24 years with running businesses in the community. Businesses are verified to ascertain if they are owned by the girls and if they are ‘viable’ based on a ‘viability assessment tool’ used by sub-recipients. Girls enrolled in EDA train for 10 days: six days for SBCC and four days for business skills training (book-keeping, customer care, finance management, business documentation, saving skills, stock-taking, basic balance sheet knowledge, etc.). The training is led by the District Commercial Officer. On completion of the training, enrolled girls can receive up to UGX 500,000, issued in two installments (UGX 300,000 in the first installment, and if there is good use of the money, the girl can receive her second/last installment of UGX 200,000). Sub-recipients have reported challenges in identifying girls with ‘viable’ businesses and, in most cases, there are fewer than the targeted number of girls supported through EDA. In key informant interviews conducted as part of this impact evaluation, some stakeholders questioned the practice of giving money directly to the girls and suggested that a more family-based approach or an approach that equips girls with hands-on skills could be better than direct money transfers. Sub-recipients occasionally reported of misuse of funds by the girls or girls who purported to own businesses that they did not but who eventually received money.
2. ***Second chance education, innovation camps and empowerment clubs***: These interventions are offered in the same way and are intended to equip out-of-school AGYW aged 10-24 years with artisanal soft skills that can help to enhance their economic independence. For second-chance education specifically, priority is given to girls who did not have a chance to complete formal education, girls who are family heads, girls who are orphans, girls who are struggling to find food, girls living with HIV, girls who are survivors of gender-based violence, girls who gave birth at an early age or who are currently pregnant, girls who were married at a young age, and girls who engage in commercial sex, among others. The training is conducted by local artisans identified by the sub-recipients and approved by TASO. Girls train for 10 days; of these, six are reserved for SBCC while four days are reserved for skills-based training. The courses offered include: bakery, liquid soap making, shoe making, candle making, craft making, and sanitary pads making. In addition, girls receive business skills in marketing, customer care, record keeping, and basic literacy and numeracy skills, among other life skills. The training is provided to girls in groups of 15-20 girls and is usually residential for second-chance and innovation camps. However, for empowerment clubs, the training is non-residential and the girls commute from their homes to the training venues. Start-up kits and or capital are given to the girls after the training to start their own business.
3. ***Sinovuyo Teens***: Sinovuyo Teens is a curriculum-based training that uses a curriculum adapted from South Africa. The full name of the curriculum is “Sinovuyo Caring Families Program for Parents and Teens”. The main purpose of this program is to improve relationships between family members to enable them communicate better. In general, the Sinovuyo Teens Program aims to increase parenting skills and confidence in raising teens, improve positive parenting behavior, help teenagers to manage their behavior including HIV risk behaviors, help families to respond better to crisis situations, and improve knowledge of referral services available in the community for voluntary counseling and testing, treatment, and care and support. Sinovuyo Teens is a non-residential, 14-session program for groups of parents and their teenagers. The groups meet weekly with two facilitators to work together on parent-teen interactions, managing stress, and building good, strong relationships. Of the 14 sessions, 10 are joint sessions in which parents and teens meet together at the same time in the same room while 4 sessions are separate sessions where teens and parents have teen-only and parent-only groups. The program targets families with parenting problems which can render a girl vulnerable to HIV, like GBV as well as very poor families in the community. The main focus is on AGYW aged 10- 19 years, who have dropped out of school but have not yet delivered children; those that are still living with their parents, and adolescent boys and young men aged 15-19 years. At the end of the training the girls are taken for soft skilling.
4. **Interventions for in-school AGYW**. These interventions were implemented by the Ministry of Education and Sports working directly with the intervention schools in each district. These interventions included distribution of school bags, exercise books, pencils and sanitary pads to individual girls within the intervention schools.
