# Supplementary material for: Impact of layered behavioral, socio-economic and school-based interventions on selected behavioral and biomarker indicators among adolescent girls and young women in Uganda
Source: PLOS Glob Public Health. 2025 Jun 24;5(6):e0004819. doi: 10.1371/journal.pgph.0004819 (PMC12186927; doi:10.1371/journal.pgph.0004819)
Supplement: S2 Text — (DOCX) [file pgph.0004819.s002.docx]

**Detailed analysis on exposure to AGYW interventions**

**Table A** shows the percentage of AGYW who participated in SBCC activities during the period 2019-2023, as conducted by the different sub-recipients (SRs). Overall, **only 4.4% (n=137) of girls in the intervention and 0.08% (n=2) of girls in the non-intervention districts reported that they participated in any SBCC activity**. More specifically, within the intervention districts, only 5% of in-school and 3.7% of out-of-school girls reported exposure to any SBCC activity supported by the SRs. In non-intervention districts, less than 1% of in-school and out of school girls reported exposure to these activities. Games including netball, football, volleyball, etc., were reported by the highest numbers of AGYW in the intervention districts at 3.4% among in-school and 1.9% among those out of school.

Table A. In the last four years, percentage of AGYW that participated in SBCC activities designed for AGYW

| **SBCC activities** | **Intervention districts (3,125)** | | **Non-Intervention**  **districts (2,332)** | |
| --- | --- | --- | --- | --- |
|  | **In-school (N=1,571)** | **Out of school (N=1,554)** | **In-school (N=1,164)** | **Out of school (N=1,168)** |
| Games (netball, football, volleyball, etc.) | 54 (3.4%) | 30 (1.9%) | 1 (6.8%) | 1 (0.2%) |
| Music, dance, and drama | 23 (1.5%) | 13 (0.8%) | - | 1 (0.2%) |
| Community sports events | 13 (0.8%) | 8 (0.5%) | - | 1 (0.2%) |
| Community outreaches/meetings | 7 (0.4%) | 3 (0.2%) | - | 1 (0.2%) |
| Essay competitions | 4 (0.3%) | 2 (0.1%) | - | - |
| ***Participated in at least one activity*** | ***79 (5.0%)*** | ***58 (3.7%)*** | ***1 (0.09%)*** | ***1 (0.09%)*** |

**Table B** shows the percentage of AGYW who received messages that required them to take action (e.g., test for HIV, enrol in HIV care, use family planning, etc.), from the different SRs, between 2019 and 2023. **Overall, 14.6% (n=458) of girls in the intervention districts and 5.4% (n=127) of girls in the non-intervention districts reported that they received any message that required them to take action**. Specifically, only 12.1% of in-school and 17.3% of those out of school, in intervention districts, were exposed to any health messages that required them to take action. In the non- intervention districts, this percentage was lower at 3.7% and 7.2% among in-school and out of school AGYW, respectively. The most reported health messages exposed to were family planning use and the need to test for HIV.

Table B. In the last 4 years, percentage of AGYW who received messages that required them to take action, delivered by the sub-recipients

| **Health messages** | **Intervention districts (3,125)** | | **Non-Intervention**  **districts (2,332)** | |
| --- | --- | --- | --- | --- |
|  | **In school (N=1,571)** | **Out of school (N=1,554)** | **In school (N=1,164)** | **Out of school (N=1,168)** |
| Screening for sexually transmitted infections | 23 (1.5%) | 11 (0.7%) | 1 (0.1%) |  |
| Testing for HIV | 49 (3.1%) | 97 (6.2%) |  |  |
| Enrolling in HIV care | 2 (0.1%) | 1 (0.1%) |  |  |
| Using family planning methods | 117 (7.4%) | 200 (12.9%) | 42 (3.6%) | 84 (7.2%) |
| Reducing number of sexual partners | 15 (1.0%) | 8 (0.5%) | 1 (0.1%) |  |
| Consistent condom-use with sexual partner | 53 (3.4%) | 22 (1.4%) |  | 1 (0.1%) |
| ***Received at least one message*** | ***190 (12.1%)*** | ***268 (17.3%)*** | ***43 (3.7%)*** | ***84 (7.2%)*** |

**Table C** shows the percentage of AGYW who received support or participated in any socio-economic or economic empowerment activities, as implemented by the SRs, between 2019 and 2023. **Overall, 15.8% (n=495) of girls in the intervention districts and 7.9% (n=185) of girls in the non-intervention districts reported that they received support or participated in any socio-economic or economic empowerment activity**. Specifically, only 15.7% of in-school and 16.0% of those out of school, in intervention districts, received support or participated in any of the socio-economic and empowerment activities from the SRs. In the non-intervention districts, this percentage was lower at 8.8% and 7.1% among in-school and out of school AGYW, respectively. The most reported socio-economic or empowerment activities, in intervention districts, included vocational skilling at 10.2% and 13.8% among in-school and out-of-school AGYW, respectively, and second chance education at 6.5% and 2.5% among in-school and out-of-school AGYW, respectively. Also, sizeable percentages of AGYW in non-intervention districts reported vocational skilling and second chance education, most probably delivered by other implementers other than the sub-recipients.

Table C. In the last 4 years, percentage that received support or participated in any of the following socio-economic or empowerment activities, implemented by sub-recipients

| **Socio-economic or empowerment activities** | **Intervention districts (3,125)** | | **Non- Intervention**  **districts (2,332)** | |
| --- | --- | --- | --- | --- |
|  | **In school AGYW (N=1,571)** | **Out of school AGYW (N=1,554)** | **In school AGYW (N=1,164)** | **Out of school AGYW (N=1,168)** |
| Second chance education | 101 (6.5%) | 39 (2.5%) | 30 (2.6%) | 12 (1%) |
| Vocational skilling | 158 (10.2%) | 211 (13.8%) | 85 (7.3%) | 70 (6.1%) |
| Enterprise development assistance (EDA) | 27 (1.8%) | 39 (2.6%) | 20 (1.7%) | 12 (1%) |
| Empowerment clubs | 31 (2%) | 21 (1.4%) | 17 (1.5%) | 5 (0.4%) |
| Dialogue meetings | 27 (1.8%) | 30 (2%) | 14 (1.2%) | 7 (0.6%) |
| Thematic radio talk shows | 13 (0.9%) | 7 (0.5%) | 13 (1.2%) | 5 (0.4%) |
| ***Received support or participated in at least one socio-economic activity*** | ***247 (15.7%)*** | ***248 (16.0%)*** | ***102 (8.8%)*** | ***83 (7.1%)*** |

**Table D** shows the percentage of in-school AGYW that received education materials provided by the Ministry of Education and Sports during the period 2019-2023. **In the intervention districts, receipt of these education materials among the sampled AGYW was at 43% while in the non-intervention districts, it was at 16.8%**. The most reported items included menstrual pads at 40.4% and 12.4% in the intervention and non-intervention districts, respectively and free exercise books at 37.9% and 10.1% in the intervention and non-intervention districts, respectively.

Table D. In the last 4 years, percent that received any of the following items, distributed by the Ministry of Education and Sports, in schools

| **Items** | **Intervention districts (N=1,571)** | **Non-Intervention**  **districts (N=1,164)** |
| --- | --- | --- |
| Free exercise books | 596 (37.9%) | 117 (10.1%) |
| Underwear | 188 (12%) | 71 (6.1%) |
| Half-petty | 183 (11.6%) | 56 (4.8%) |
| Menstrual pads | 634 (40.4%) | 144 (12.4%) |
| **Received at least one item** | **676 (43.0%)** | **195 (16.8%)** |

When AGYW who reported that they received at least one educational material (i.e., 871) were asked how often they received these materials in a typical calendar year, 43.5% of girls in the intervention districts versus 50.4% of girls in the non-intervention districts reported that they received them once in a typical calendar year (**Figure SF2_1**). However, nearly 44% of girls in the intervention districts reported that they received the items once every school term as opposed to 28.2% of girls in the non-intervention districts. Only 4.5% of girls in the intervention and 7.7% of girls in the non-intervention districts reported receiving the materials twice every school term. **The fact that girls in the non-intervention districts also reported receiving the same materials as those in the intervention districts possibly confirms the fact that there were other implementing partners that targeted in-school girls** in the non-intervention districts with the same materials. The effect of these other interventions is to dilute the effect of the interventions implemented in the intervention districts as no marked difference can be seen in terms of behavioral or biomarker indicators between girls in the two types of districts.

Figure A. Frequency of receipt of education materials among girls in the intervention vs. non-intervention districts

AGYW who reported that they received menstrual pads (i.e., 634 in the intervention districts and 114 girls in the non-intervention districts) were asked how often they received free menstruation pads during a typical school term. As shown in **Figure B**, only 43.4% of girls in the intervention and 35.4% of girls in the non-intervention districts reported receiving free menstrual pads once during the school term. Ten per cent of the girls in the intervention districts reported receiving the pads monthly while 5.5% reported that they received them once every two months.

Figure B. Frequency of receipt of free menstrual pads while at school during the school term
