# Supplementary material for: Impact of layered behavioral, socio-economic and school-based interventions on selected behavioral and biomarker indicators among adolescent girls and young women in Uganda
Source: PLOS Glob Public Health. 2025 Jun 24;5(6):e0004819. doi: 10.1371/journal.pgph.0004819 (PMC12186927; doi:10.1371/journal.pgph.0004819)
Supplement: Attachment — Submitted filename: Paper Review.docx [file pgph.0004819.s003.docx]

Thanks to the authors of this paper, they really did a great work and I am honestly impressed.

Just a few comments to check:

**Comments**

***Measurable variables***

A (ii)

FGDs and key informant interviews since parents and guardians were involved an acuity of the intervention?

Did the author consider the risky sexual behaviours of those girls out of school?

Referral and linkage of those tested with an STI and follow-up?

Results

21 Reporting of results been divided into two, but there is also “C”?

**Recommendation**

Clearly state the inclusion criteria

Check the denominators in the tables and verify within the description of table.

Would the author consider the number of children as a vital variable for the intervention?
