# Supplementary material for: Impact of layered behavioral, socio-economic and school-based interventions on selected behavioral and biomarker indicators among adolescent girls and young women in Uganda
Source: PLOS Glob Public Health. 2025 Jun 24;5(6):e0004819. doi: 10.1371/journal.pgph.0004819 (PMC12186927; doi:10.1371/journal.pgph.0004819)
Supplement: Attachment — Submitted filename: Response_to_Reviewers.doc [file pgph.0004819.s005.doc]

**POINT-BY-POINT RESPONSE TO THE REVIEWERS’ COMMENTS**

**Reviewer #1**

General comments

The study investigated the impact of layered behavioral, socio-economic and school-based interventions on selected behavioral and biomarker indicators among adolescent girls and young women in Uganda. A comprehensive background to the problem has been given. It suggests that whereas adolescent girls and young women are at an elevated risk of HIV infection globally, East and Southern African countries continue to be more vulnerable, including Uganda. Factors such as inequalities, discrimination and poverty deny many women and adolescent girls’ economic autonomy,deprive them of control over their sexual lives, and expose them to ongoing HIV risk. Again, other identified drivers for HIV infection include intimate partner violence where women are unable to negotiate for safe sex with the abusive partner, child sexual abuse, and early sex debut which also contributes to teenage pregnancy. The study further urges that exposing adolescent girls and young women to multiple HIV prevention interventions such as cash transfer schemes or education subsidies, skills training programs and linking comprehensive sexuality education with sexual and reproductive health services, etc. have the potential of reducing the risks to HIV infection by decreasing the involvement in risky sexual behaviour and decreasing teenage motherhood. The study is relevant and has a clearly stated objective. The write up is good except for a few editorial issues as outlined below.

**Response: We thank the reviewer for the great summary of our paper and the compliments.**

MAJOR ISSUES

Introduction
a. The background is well stated. However, the authors need to provide a brief description of why the study is important. What its public health significance?

**Response: We have provided a brief on the public health significance of the study in the revised version of the paper – see page 4, lines 31-34.**

Methodology
a. Line 23, pg. 4: It is stated that ‘‘Based on the program’s Theory of Change (Figure 2) …’’ The authors introduce to the readers a theory the study used. However, it would be good if the authors talk about briefly what the theory of change is about and its core principles before showing us how it was applied in their study.

**Response: We have provided a brief on what the theory of change is all about, so that our readers can connect well and understand the study’s theory of change – see page 6, lines 23-30.**

b. On data analysis section, it would be helpful if the authors indicate first the kind of the analytical methods used in the study (statistical tests) before explaining the steps taken in the analysis. For example, in the findings, tables 5, 7, and 9, confidence intervals are stated which can be associated with specific types of statistical analyses. Again, they can state in a sentence or two the justification why those methods were chosen.

**Response: We used the proportion command in STATA (version 16.0) to compute the 95% confidence intervals associated with the biomarker estimates in Tables 5, 7 and 9. We have amended the data analysis subsection to cater for this clarification – see page 13, lines 2-3.**

Discussion
a. Even though the authors showed how the theory of change was used for AGYW interventions, the discussions it is not shown how the findings are related to the theory.

**Response: Given the modest impact of the interventions; the program’s theory of change was generalized not realized. Thus, we were unable to link any results to the theory of change. We have made this clarification on page 21, lines 18-20.**

b. The discussion of findings needs strengthening. Although the authors do well to explain what their findings mean in the context of their research, more is needed with regards to comparing the current findings with previous research. Additional literature on the current four used in the discussion is necessary.

**Response: We have added literature that compares our findings to previous findings, as suggested by the reviewer. See lines 19-29 on page 22, for details.**

c. Only one recommendation is provided even though important findings are identified which can influence recommendations for policy, practice, and research. This has to be improved. Some of such recommendations are stated in the discussion of findings and hence need to be teased out and brought to the rightful place. Again, since this is a crucial topic that has been studied, it would be good to suggest the bodies that will need to enforce the recommendations provided for the study to become relevant and contribute to the wellbeing of the participants. For example, is there a way the government, especially targeted ministries like the Ministry of health, policy makers, funders, schools, health care professionals, and other partners can come in to support and how?

**Response: Study findings boil down to one thing: that the interventions had a modest impact on the targeted behavioral and biomarker indicators. We have tried to provide probable reasons why we think these interventions did not yield the expected impact, as envisioned through the AGYW program’s Theory of Change. However, since we do not have data on some of the probable reasons (some of them are based on our observations; others arise from our program evaluation experience), we are not comfortable making recommendations beyond the data that we have. This is the same reason why we did not specifically point out areas that respective implementing agencies, including government ministries, policymakers and funders could take on to improve the performance of the interventions. Nevertheless, we have included a generic recommendation to all implementers: “*In future, it will be important for program implementers to pay a close attention to not only the intensity of intervention delivery but also to the fidelity of implementation and duration of exposure to improve the impact of AGYW interventions on the selected behavioral and biomarker indicators*.” (see page 21, lines 20-22).**

MINOR ISSUES

Abstract
a. Line 21-22, pg. ii: The abstract is good. However, authors need to provide absolute numbers or percentages/proportions for some statements in the findings section for a clear picture. For example, half of the AGYW were in school (what would the readers consider as half in relation to your study population); about two-thirds were single/never married.

**Response: We have revised the abstract to clarify the language as needed, and have provided absolute numbers or percentages as needed, to improve clarity (See page 2, “abstract”, for details). We have also reduced the text in response to a comment from reviewer #2.**

b. Line 32, pg. ii: ‘‘… HIV syphilis was lower…’’ Check out for these words and correct accordingly

**Response: We thank the reviewer for pointing out this minor error. We have now corrected it to read, “Syphilis prevalence ...” See page 2, line 23.**

Methodology
a. Line 29-32, pg. 10: the authors seem to state their objectives for the study in the data analysis section. If yes, then these need to be clearly stated in the background of the study and briefly mentioned here for reinforcement.

**Response: As advised, we have added the study objectives to the last paragraph of the background section, see page 5, lines 3-6, for details.**

Discussion
a. Line 13, pg. 19 ‘‘ b) the period of exposure may have too short to allow for behavior change to occur’’ add the word ‘‘been’’ between ‘‘have and short’’.

**Response: We thank the reviewer for his keen eye. We have added word “been” that had been missed from the original version – see page 21, line 13.**

**Reviewer #2**

**Title: Impact of layered behavioral, socio-economic and school-based interventions on selected behavioral and biomarker indicators among adolescent girls and young women in Uganda**

However the title is somehow different from the stated objective of "This paper reports the findings of an impact evaluation of the impact of behavioral, socio-structural and educational interventions targeting in- and out-of-school AGYW on the triple burden of HIV, teenage pregnancy and intimate partner violence among AGYW in Uganda" stated in from line 34 page 2 to lines 2-3 page 3.

**Response: The paper title has now been harmonized with the overall objective of the impact evaluation, as stated on page 5, lines 1-3.**

Abstract:
With a word count of 533 is well above the maximum 300 words allowed.
Abstract not expectd to have subsections acording to guidelines given.

**Response: We have removed the subsections in the abstract as guided. We have also reduced the words in the abstract to 389 words. Please note that we exceeded the 300 word-limit mark because the journal’s manuscript submission portal allows for abstracts of up to 500 words; this gave us the leeway to exceed the 300 word-limit mark that is referred to in the authors’ guidelines. Indeed, we have checked several published journal articles and realized that their abstracts have varying word limits, possibly because of the flexibility to exceed the 300 word-limit as indicated in the journal’s submission portal. In line with this flexibility but also taking into consideration the reviewer’s comment on the abstract word-count, we have removed all the details about the interventions – which has pushed down the number of words to 401. We note that this is still above the 300 word-limit mark that is referred to in the comment – but since there is some flexibility on the word-limit, we will await the Editor’s guidance on the next steps on this issue.**

Introduction:
This statement in lines 9-10 ".....HIV prevalence among adolescent girls (aged 12-19 years) increased from 4.2% to 5.7% compared to an increase from 1.8% to 4.5% among their male counterparts" contradicts what is stated in lines 6-7 "...15% of all new infections occur among adolescents aged 10–19 years, with 83% of adolescent infections occurring in girls."

**Response: The two statements come from two different sources; one from the UNAIDS report (2024) and the other from population-based surveys conducted in Botswana between 2008 and 2017. We did not use the second statement as proof of the first one but to emphasize that girls remain more at risk than boys. Thus, the percentages cited from the Botswana survey are not intended to confirm that 83% of new HIV infections occur in girls but to emphasize that HIV prevalence in girls remains higher than HIV prevalence in boys. We have edited these statements to improve clarity (see page 3, lines 7-10, for details). We hope the edits have helped to clarify the purpose of the second statement.**

Methods;
Lines 31-33 the statatement "In the 2023 survey specifically, data were also collected from the non-intervention villages and schools to aid in the computation of the difference-in-difference estimates during the assessment of the impact of the interventions on the behavioral and biomarker indicators (see ‘Measurement of variables’ below)" does it imply that in the 2018 survey data were not collected from non-intervention areas.

**Response: We thank the reviewer for pointing this out. We have edited the statement to refer to the fact that in 2023, surveys were conducted in the same villages/schools as in the 2018 survey to allow for the estimation of the difference-in-difference estimates. The new sentence now reads: “*In the 2023 survey, data were collected from the same villages and schools as in the 2018 survey to aid in the computation of the difference-in-difference estimates during the assessment of the impact of the interventions on the behavioral and biomarker indicators*” (see page 9, lines 1-4).**

Discussion:
In lines 19-20 & 28-29 the authors make reference to "qualitative interviews" that were not presented in the results section.

**Response: While we conducted some qualitative interviews, we did not present our qualitative methods or findings in the current paper. Thus, we agree that it would be inappropriate to refer to qualitative interviews in the “Discussion” section (for the first time) when we did not present any prior qualitative findings. For this reason, and given that we did not present our qualitative methods or qualitative findings prior to getting to the “Discussion” section, we have opted to edit out all references to “qualitative interviews” and instead refer to our interactions and observations during the impact evaluation (e.g., see lines 6-7, page 23). We hope that this helps to address this comment.**

The study period includes the period 2020-2023 when there was COVID 19 pandemic disruptions yet the authors do not mention its effect in the discussion/limitations.

**Response: We have made reference to the effect of COVID-19 lockdown during the implementation period which may have affected the implementation of interventions targeting AGYW (page 23, lines 21-24). However, since we did not collect data on this issue, we have acknowledged the lack of data on the impact of COVID-19 on the implementation of interventions targeting AGYW as a study limitation.**

**Reviewer #3**

Thanks to the authors of this paper, they really did a great work and I am honestly impressed.

Just a few comments to check:

**Comments**

***Measurable variables***

1. A (ii) FGDs and key informant interviews since parents and guardians were involved an acuity of the intervention?

**Response: This comment is not clear to us. However, taken from face value, we would like to clarify that we did not conduct any interviews with parents or guardians as the impact evaluation was meant to assess behavioural and biomarker indicators among the girls reached with the interventions.**

2. Did the author consider the risky sexual behaviours of those girls out of school?

**Response: We used the same questionnaire for both in- and out-of-school AGYW. So, yes, we considered the risk behaviours of both in- and out-of-school AGYW.**

3. Referral and linkage of those tested with an STI and follow-up?

**Response: As indicated on page 10, lines 22-25, we had a plan for those who tested positive or negative for both syphilis and HIV, working closely with the nearest health facility.**

Results

4. 21 Reporting of results been divided into two, but there is also “C”?

**Response: This comment is not clear to us. However, if this refers to the categorization of results based on: a) exposure status, and b) impact of the interventions in the intervention/non-intervention districts or schools, then, we would like to clarify that the category reporting on the impact of interventions includes: (i) impact in the intervention/non-intervention districts and (ii) intervention/non-intervention schools; thereby catering for all the levels of results that we intended to provide in the paper. We hope this helps to clarify the reporting of results.**

**Recommendation**

5. Clearly state the inclusion criteria

**Response: The impact evaluation targeted girls aged 10-24 years because this was the age-range that was targeted by the interventions, see page 5, lines 21-22.**

6. Check the denominators in the tables and verify within the description of table.

**Response: We have checked the tables and we think that the description provided for each table is consistent with the numbers indicated in each table. Note that in some instances, the numbers in the tables were combined during the reporting (e.g., ‘*very often*’ and ‘*often*’ in the reporting of exposure to health messages in Tables 2 and 3) but even then, a simple computation shows that the two for both ‘*very often*’ and ‘*often*’ is the same as reported in the text.**

7. Would the author consider the number of children as a vital variable for the intervention?

**Response: This comment is not clear to us. However, taking it on face value, we can safely report that we did not collect data on the number of children. We will be glad to provide a more elaborate response to a more specific comment, as needed.**
