# Supplementary material for: Impact of layered behavioral, socio-economic and school-based interventions on selected behavioral and biomarker indicators among adolescent girls and young women in Uganda
Source: PLOS Glob Public Health. 2025 Jun 24;5(6):e0004819. doi: 10.1371/journal.pgph.0004819 (PMC12186927; doi:10.1371/journal.pgph.0004819)
Supplement: Attachment — Submitted filename: Response to Reviewer_Comments_May 29, 2025.doc [file pgph.0004819.s006.doc]

**Response to Reviewer’s Comments**

1. The study covered the years 2018 - 2023 and this include the COVID19 pandemic period 2020 - 2022 marked with lockdowns and various restrictions yet the authors have conveniently avoided discussing its effect on the interventions an results.

**Response: We have included a paragraph in which we discuss the likely impact of the COVID-19 lockdowns on the interventions and the observed lack of impact of the interventions on behavioral and biomarker indicators that we assessed. See lines 4-13, page 22.**

2. The survey in 2018 was conducted in 20 districts while the one in 2023 was carried out in 14 districts. An explanation ought to e given as to why the 6 districts were not included in 2023.

**Response: We have provided an explanation as to why six of the initially surveyed districts (in 2018) were not resurveyed in 2023. See lines 30-32, page 5.**

Specific comments:

1. Abstract - Line 32 the phrase "HIV Syphillis" needs to be corrected.

**Response: We were not able to identify any statement with ‘HIV syphillis’ in the abstract – since this was already corrected in our initial revision.**

2. Introduction - phrase in lines 9 - 10 appear to be contradictory to what is stated in lines 6 -7.

**Response: Thanks for the comment. However, we were unable to detect any contradictions. All indications point to the fact that adolescent girls and young women are more prone to HIV infection than their male counterparts. We will be glad if the reviewer can point out any contradictions that they noted so we can address them in a more appropriate manner.**
